# Supplementary material for: TFAP2A links drug resistance to antitumor immunity
Source: bioRxiv. 2026 Jul 10:2026.07.08.735861. Preprint. [Version 1] doi: 10.64898/2026.07.08.735861 (PMC13370983; doi:10.64898/2026.07.08.735861)
Supplement: 1 [file NIHPP2026.07.08.735861v1-supplement-1.pdf]

bioRxiv preprint doi: https://doi.org/10.64898/2026.07.08.735861; this version posted July 10, 2026. The copyright holder for this preprint (which was not certified by peer review) is the author/funder, who has granted bioRxiv a license to display the preprint in perpetuity. It is made available under a CC-BY 4.0 International license.

Supplementary Figures and legends.

Supplementary Figure 1

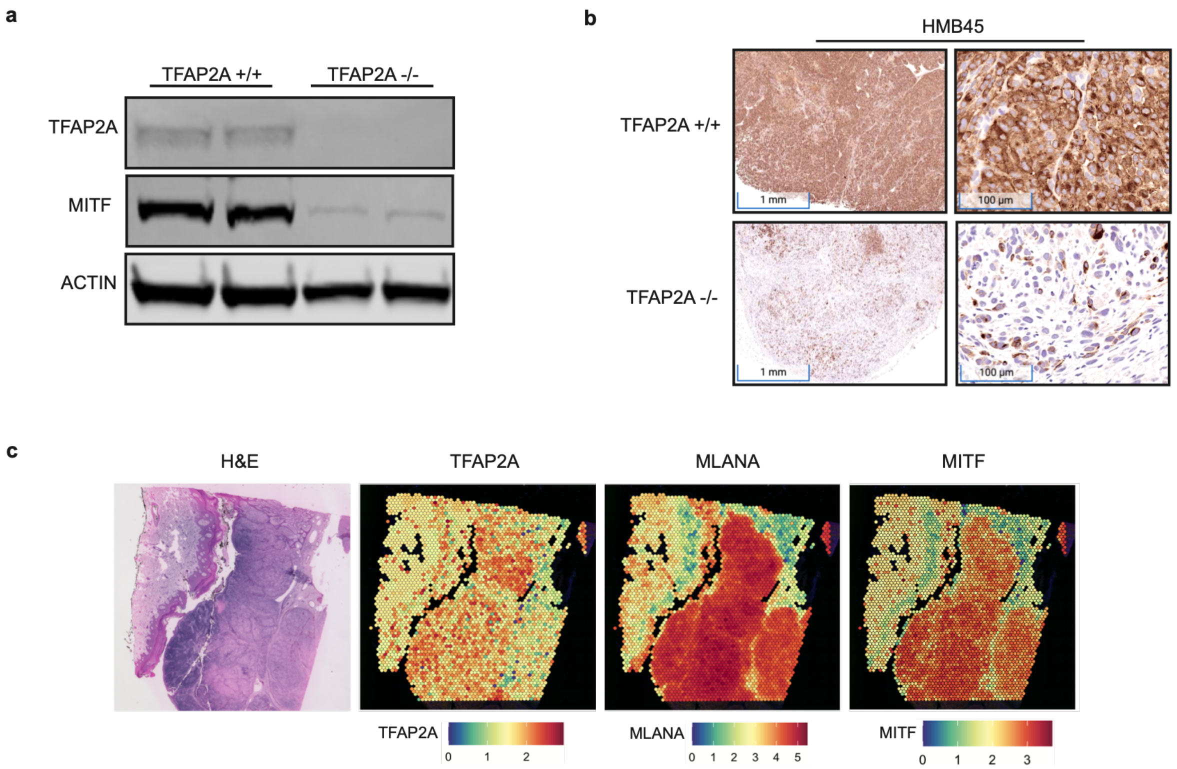

Supplementary Figure 1. **TFAP2A regulates pigmentation genes**. (**a**) Western blot shows that MITF expression is downregulated in TFAP2A-KO cells. (**b**) TFAP2A-KO melanomas show less pigmentation marker, HMB45. (**c**) Correlation analysis from public spatial single cell RNA sequencing dataset reveals that TFAP2A spatially correlates well with the expression of MLANA and MITF, which are the pigmentation molecules.

bioRxiv preprint doi: https://doi.org/10.64898/2026.07.08.735861; this version posted July 10, 2026. The copyright holder for this preprint (which was not certified by peer review) is the author/funder, who has granted bioRxiv a license to display the preprint in perpetuity. It is made available under a CC-BY 4.0 International license.

Supplementary Figure 2

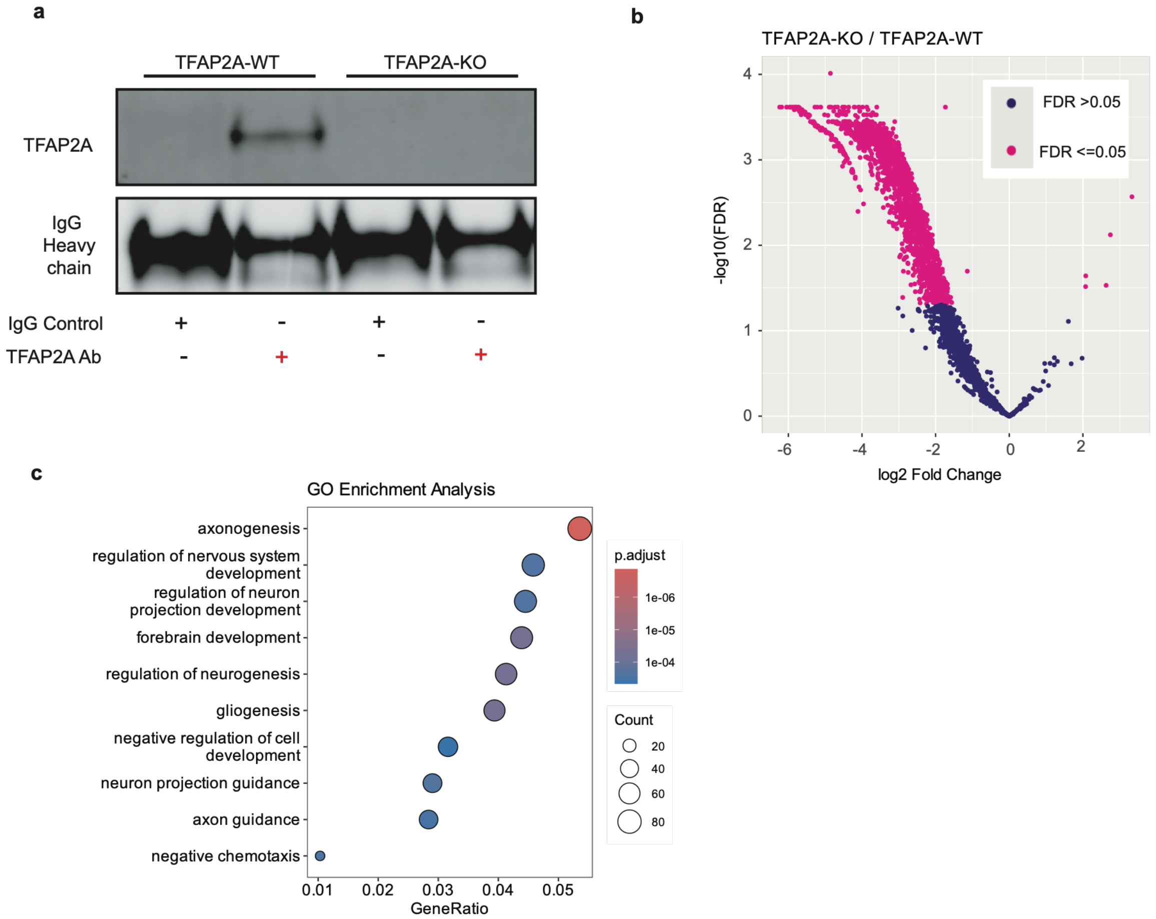

Supplementary Figure 2. **ChIP-seq to identify direct transcriptional targets of TFAP2A**. (**a**) Co-Immunoprecipitation (CoIP) shows antibody against TFAP2A successfully pull down the target protein TFAP2A. (**b**) ChIP-seq using the antibody in (a) successfully pulled down gene fragments which are annotated and presented as volcano plot. (**c**) GSEA analysis using the annotated genes from (b) show TFAP2A targets are enriched in neurogenesis pathways.

bioRxiv preprint doi: https://doi.org/10.64898/2026.07.08.735861; this version posted July 10, 2026. The copyright holder for this preprint (which was not certified by peer review) is the author/funder, who has granted bioRxiv a license to display the preprint in perpetuity. It is made available under a CC-BY 4.0 International license.

Supplementary Figure 3

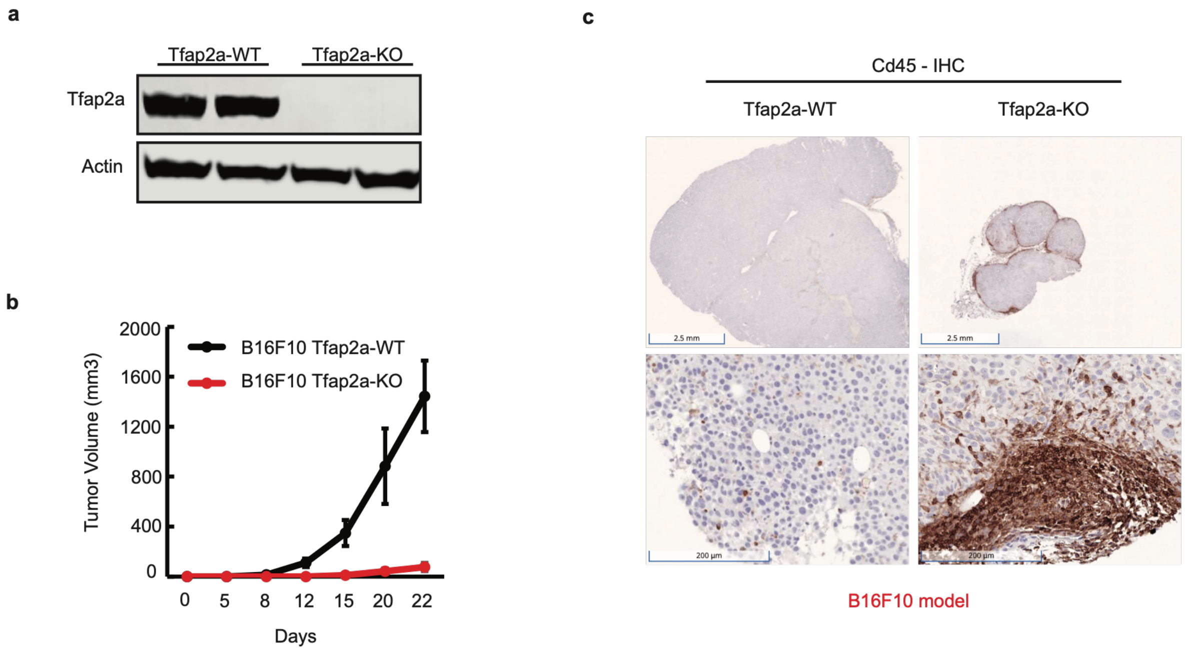

Supplementary Figure 3. **Tfap2a knockout impairs tumor growth and promotes formation of immune clusters in B16F10 melanoma model.** (**a**) Western blot confirms the protein loss of Tfap2a in B16F10 knockout cells. (**b**) Tumor growth curve of B16F10-Tfap2a-WT or B16F10- Tfap2a-KO melanomas in C57BL/6J mice (n=5). (**c**) Immunohistochemistry (IHC) staining of Cd3+ T cells in Tfap2a-WT or Tfap2a-KO melanomas. Brown color indicates the positive staining of Cd3+ cells.

bioRxiv preprint doi: https://doi.org/10.64898/2026.07.08.735861; this version posted July 10, 2026. The copyright holder for this preprint (which was not certified by peer review) is the author/funder, who has granted bioRxiv a license to display the preprint in perpetuity. It is made available under a CC-BY 4.0 International license.

Supplementary Figure 4

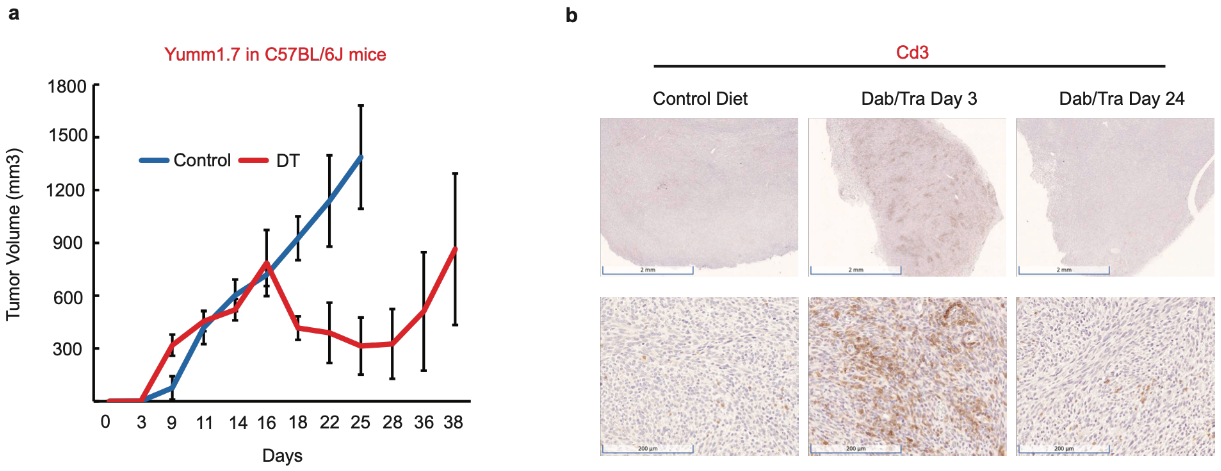

Supplementary Figure 4. **Recurrent melanomas show immune-cold feature**. (**a**) Yumm1.7 cells were subcutaneously injected into NSG mice for tumor growth curve. Dabrafenib/Trametinib (D/T) chow or control chow were given on day 16. Red curve shows the Yumm1.7 melanomas respond to BRAF/MEK inhibitors (D/T) initially, shrink robustly and quickly develop relapse. (**b**) Immunohistochemistry (IHC) staining of Cd3+ T cells in Yumm1.7 melanomas treated with Dabrafenib/Trametinib (Dab/Tra) for different period of times (Day 0, Day 3 and Dady 24). Brown color indicates the positive staining of Cd3+ cells.

bioRxiv preprint doi: https://doi.org/10.64898/2026.07.08.735861; this version posted July 10, 2026. The copyright holder for this preprint (which was not certified by peer review) is the author/funder, who has granted bioRxiv a license to display the preprint in perpetuity. It is made available under a CC-BY 4.0 International license.

Supplementary Figure 5

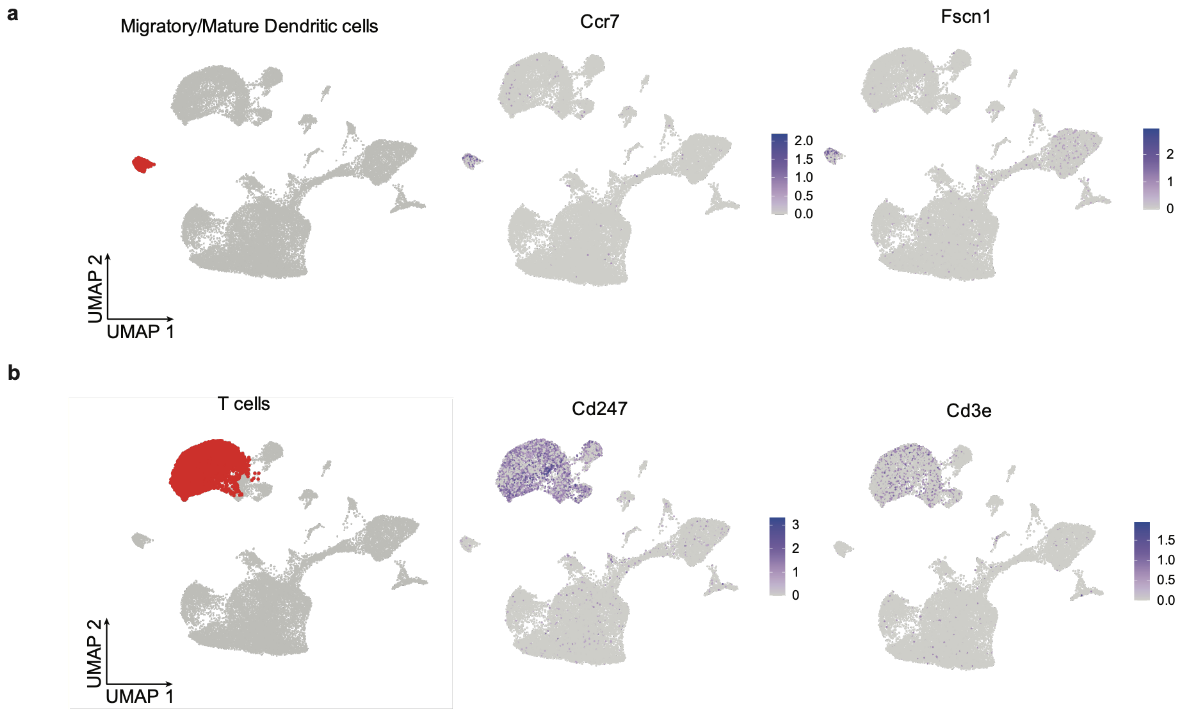

Supplementary Figure 5. **Sing cell RNA-seq annotation of Dendritic cells and T cells**. (**a**) Dendritic cell subset within Yumm1.7 melanomas. Ccr7 and Fscn1 are markers for mature dendritic cells. (**b**) T cell subset. Cd247 and Cd3e are markers for T cells.
